# The Role of Sources of Informal Care on Depressive Symptoms in Older Mexican Americans

**DOI:** 10.1177/30495334251395355

**Published:** 2025-11-20

**Authors:** Aymun Razzak, Anna Bokun, Phillip Cantu

**Affiliations:** 1 University of Texas Medical Branch, Galveston, USA; 2 UT Austin, TX, USA

**Keywords:** long-term care, aging, depression, disability, mental health, caregiving, and management

## Abstract

**Background:**

Research on informal caregiving has focused on caregivers, with less attention paid to how caregiving relationships impact care recipients’ mental health. Understanding how different sources of care influence depressive symptoms is crucial for designing more effective, culturally appropriate interventions for older adults.

**Methods:**

We used data from the seventh wave of the Hispanic Established Populations for the Epidemiologic Study of the Elderly (H-EPESE; *n* = 760). We examined the association between depressive symptoms and sources of care for Instrumental Activities of Daily Living (IADL) disability in older Mexican Americans aged 80+. Depressive symptoms were measured using the Center for Epidemiological Studies Depression (CESD) scale. Ordinary least squares (OLS) regression was used to model the relationship between depressive symptoms and care sources, adjusting for demographic and health covariates.

**Results:**

The average CESD score among care recipients was 10.71. Sources of care include 45% self-care, 67% family care, and 22% non-family care. Regression analysis showed that self-care significantly reduced depressive symptoms (−4.41, *p* ≤ .01), family care reduced symptoms (−1.98, *p* ≤ .01), while non-family care increased depressive symptoms (4.03, *p* ≤ .01).

**Conclusion:**

Findings highlight the importance of family-centered caregiving and suggest key policy implications: implementation of caregiver skills training, peer support groups, and targeted mental health resources.

## Introduction

As of 2023, over 38 million adults in the United States have served as informal caregivers, providing support to adults with limitations in daily functioning (Bureau of Labor Statistics, 2023). Informal caregiving is defined as unpaid assistance with daily activities provided to individuals with chronic illness or disability; it has extensively examined caregivers’ mental health outcomes, and less attention has been paid to how these care relationships affect care recipients (Roth et al., 2015). Instrumental Activities of Daily Living (IADL) care in particular deserves attention, as individuals in the early stages of health-decline need the most assistance yet show the greatest resistance to accepting help (Verbrugge & Jette, 1994). Early recognition and intervention in IADL disabilities is crucial, as it can significantly influence the progression of the caregiving relationship and improve outcomes for both caregivers and care recipients (Lin & Wu, 2011). While care for older adults can come from paid and unpaid sources, informal unpaid caregivers make up the majority of help provided to older adults (Cantu et al., 2024). We use the term ‘caregiver’ to refer primarily to unpaid, informal caregivers, usually family members, while ‘care worker’ is reserved for paid individuals providing formal or semi-formal care.

Chronic conditions affect multiple domains of daily life, from Activities of Daily Living (ADLs), like basic personal care, to more complex household management (IADLs) and employment. While ADLs such as eating, toileting, transferring, dressing, and bathing are essential for survival, IADLs including meal preparation, housework, financial management, telephone use, and shopping, are crucial for maintaining meaningful social connections and independence (Verbrugge & Jette, 1994). The ability to perform IADLs significantly impacts quality of life and mental health, making them essential for independent living. Older adults living with disability are particularly vulnerable to depressive symptoms due to the combined effects of chronic disease burden, social isolation, and functional decline. This vulnerability is further explained by the Disablement Process model, which describes how progressive functional limitations can erode independence and role identity, ultimately increasing the risk for poor mental health (Kennedy et al., 1990; Verbrugge & Jette, 1994).

Older adults often rely on informal caregivers to remain in their communities and avoid nursing home placement (Thomeer et al., 2015). This reliance on informal care is particularly pronounced among Mexican Americans, who are less likely than non-Hispanic Whites to utilize formal caregiving services or nursing homes (Angel et al., 2004). In the context of older Mexican Americans, caregiving is most often carried out by family members rather than paid professionals. This reflects not only cultural expectations around family care but also practical barriers such as cost, language access, underinsurance, poverty, and immigration-related challenges that limit access to formal dementia services (Aranda et al., 2023). Mexican American expectations of female sacrifice and familial piety, or ‘Marianismo’ build an expectation among older adults that care in late life should be provided by family members (Mendez-Luck & Anthony, 2016). Furthermore, aging Mexican Americans make clear early in the aging process, (in their mid- 60s) that their preferences for aging are to remain in their homes and specifically to receive necessary care from family members (Angel et al., 2004). While much is known about preferences for aging and provision of care for Mexican Americans, little is known about how alignment of those expectations affects older adults’ outcomes. Our study addresses this gap by examining how different sources of care for disabilities relate to depressive symptoms among older Mexican Americans.

Aging can be an isolating experience, particularly when accompanied by physical and cognitive decline. Indeed, declining health and increasing disability are primary factors in the emergence of depression, often overshadowing the protective effects of social support and other life circumstances (Kennedy et al., 1990). While research shows that individuals with strong support systems cope better with stressful events than those with limited support (Greenglass et al., 2006), the caregiving relationship itself can become strained over time, with potentially negative interactions between older adults and their caregivers (Cantu & Aranda, 2023). Stressful interactions may reduce the motivation of disabled or depressed older adults to engage in recovery, further aggravating debilitating symptoms (Lin & Wu, 2011). Caregiving can be emotionally and physically demanding, especially when it involves intimate tasks like bathing, dressing, or toileting. Despite the intensity of this work, it is often undervalued both within families and in society and typically falls on women (Mendez-Luck & Anthony, 2016). Because it’s rarely seen as skilled labor, caregivers are often left without adequate support, increasing their financial and emotional vulnerability. Yet many still describe meaningful and positive relationships with the people they care for, reflecting how caregiving can be both challenging and deeply rewarding (Cantu & Aranda, 2023). Thus, while receiving care provides essential benefits, it may paradoxically contribute to increased depressive symptoms and feelings of burden among care recipients.

Informal caregiving, in particular, has been associated with negative effects on caregivers, especially in the face of life stressors that heighten rates of depressive symptoms. Elevated depressive symptoms, in turn, have been linked to higher mortality rates among older adults, with depressed individuals experiencing mortality rates two to four times higher than their non-depressed counterparts. These depressive symptoms are prevalent among older Mexican Americans (Black & Markides, 1999), raising the question of whether older Mexican Americans who receive informal care may be particularly susceptible to depressive symptoms. In line with previous research, we use a modified version of the Chronic Care model to examine how sources of care are associated with depression outcomes among older adults. This model identifies four key domains of informal and formal support for older adults: household resources, care support, community resources, and health systems (Angel et al., 2022). Although the original model was developed to examine care for older adults with dementia, we adapt this conceptual framework to examine the broader care needs of older adults.

Prior research suggests that older Mexican Americans often experience intertwined trajectories of cognitive decline and diminishing emotional or instrumental support from family members, which may accelerate psychological distress when caregiving is inadequate or absent (S. M. Rote et al., 2020). These overlapping patterns highlight the importance of understanding how the structure and source of caregiving influence depressive symptoms in this population. Our study focuses specifically on how household resources and care support contribute to depression outcomes among older adults, recognizing that caregiving arrangements vary considerably based on family dynamics, ethnicity, age, gender, and immigration status.

## Household and Individual Resources

### Caregiving Assistance

While extensive research documents the impact of caregiving on caregivers’ mental health, considerably less attention has been paid to how caregiving affects care recipients’ mental health outcomes. Previous work has highlighted how caregivers influence care recipients’ mortality, neuropsychological symptoms, and depression. For instance, higher levels of caregiver burden are associated with increased mortality rates among care recipients (Schulz et al., 2021), and poor caregiver mental health contributes to higher care recipient mortality (Lwi et al., 2017). Caregivers also play a crucial role in the progression of care recipients’ neuropsychological symptoms. Research indicates that closer caregiver-care-recipient relationships are associated with fewer affective symptoms and smaller increases in psychosis, agitation, and aggression over time (Vernon et al., 2019). Moreover, caregivers’ perceptions of the caregiving relationships further shape the mental and physical health of care recipients. While caregivers’ mental health is not directly associated with recipients’ depression and anxiety, care recipients whose caregivers report benefits of caregiving show lower odds of depression. Conversely, when caregivers experience high levels of burden and stress, their care recipients face higher odds of disability (Pristavec, 2019).

Research shows a complex paradox in caregiving arrangements, indicating that while consistent informal caregiving by family members generally reduces institutionalization and mortality risks among older adults, recipients who experience sporadic or inconsistent support face higher risks for both outcomes (Jordan et al., 2024). These findings highlight the importance of stable and continuous caregiving. Additionally, self-directed care is linked with reduced odds of institutionalization and mortality, suggesting that autonomy in care decisions may offer unique benefits. Together, these results emphasize that both continuity and individual agency in caregiving play crucial roles in optimizing long-term health outcomes.

Additionally, studies have shown that disability and depressive symptoms reinforce one another, with individuals who experience higher levels of depression often requiring increased support (Lin & Wu, 2011). Informal caregivers, particularly women, bear a substantial emotional and physical burden, highlighting the need for policy interventions to support informal caregivers and reduce the negative impacts of caregiving on their well-being.

This study contributes to the growing literature on the role of caregiving in older adults’ health outcomes by establishing the association between source of care for disability and depression in the Mexican American population. As a group that relies on and expects more informal care than other segments of the U.S. older adult population, understanding the role of informal care in depressive symptoms is imperative.

## Methods

### Data Source

Data for this study come from the seventh wave of the Hispanic Established Populations for the Epidemiologic Study of the Elderly (H-EPESE; 2010/2011), a longitudinal study of Mexican American older adults residing in five southwestern states: Texas, New Mexico, Colorado, Arizona, and California. The care recipient is asked to name a caregiver who is primarily responsible for their care to complete an informant survey. In 2010/2011 (Wave 7), a total of 1,078 subjects aged 80 and over were interviewed. During this wave, the elderly participants were asked to provide the name and contact information of the person they were ‘closest to’ or ‘depended on the most for help’. These informant caregivers (*n* = 925) were then interviewed about the health, finances, living arrangements, and general circumstances of the older care recipients. Data collection for the H-EPESE is covered by the University of Texas Medical Branch IRB protocol (available upon request). All data were de-identified and obtained from publicly available secondary datasets with no direct contact with participants.

### Sample

Our sample includes older adults aged 80+ with a family caregiver (*n* = 760). Family members include spouses (*n* = 63), children (*n* = 559), and other extended family members (*n* = 138). We omit informants who were paid employees (*n* = 42) or other non-family members (*n* = 34) and those missing information on depressive symptoms (*n* = 89). We limit our sample to respondents with spouse, child, or other family member informants due to data limitations; extended interviews on respondent care delivery were asked neither of non-family members or paid employees. Our final analytic sample consists of 760 older adults with non-missing information.

### Dependent Variable (Depressive Symptoms of the Care Recipient)

Depressive symptoms of the care recipient are measured using the Center for Epidemiologic Studies Depression (CESD) scale, a widely validated instrument that measures the presence and severity of depressive symptoms. The scale consists of 20 items evaluating symptoms experienced in the previous week, with total scores ranging from 0 to 60, where greater scores indicate greater symptom severity. The CESD has been extensively used in research with both older adults and family caregivers of older Mexican Americans (S. Rote et al., 2015). The CESD has high reliability and a Cronbach’s alpha of .89 in this sample.

### Independent Variable (Sources of Care)

The key independent variable is the source of care for recipients. The caregivers are asked to identify who is primarily responsible for the care recipient’s household tasks, selecting from four categories: self-care (care recipient themselves), the informants themselves, other family members, and non-family member care. Our sample is restricted to respondents with family member informants, and we combine ‘informant care’ and ‘other family member care’ into a single ‘family care’ category. While informants can report multiple sources of care, the questionnaire structure does not allow us to distinguish between specific types of family caregivers (e.g., spouses, children, or grandchildren).

### Covariates

Our analysis controls for factors associated with care recipients’ depressive symptoms, including demographic characteristics (age, sex, nativity status) and level of IADL disability (measured as a total count of IADL limitations). Age is calculated as the number of years at the time of the interview based on the date of the interview and the birthdate of the respondent. Sex of respondents is self-reported as baseline. Nativity status is self-reported as U.S. born or foreign-born. Information on IADL disabilities is reported by caregiver informants. Caregivers assess whether care recipients ‘need help from you or another person or special equipment, or a device across 10 activities: using the telephone, traveling alone, grocery shopping, meal preparation, light housework, medication management, handling money, heavy housework, navigating stairs, and walking a half-mile. Notably, due to the question structure and varying disability levels, some respondents classified as having IADL disabilities are still reported as primarily responsible for their household tasks.

### Analytic Plan

We examine the relationship between depressive symptoms and sources of care using ordinary least squares (OLS) regression. In Model 1, we control for demographic factors associated with depressive symptoms and sources of care. Model 2 adds controls for the number of IADL disabilities to assess if the relationship between depressive symptoms and sources of care is mediated by the level of disability. Finally, in Model 3, we limit our sample to older adults with at least 1 IADL disability, testing the robustness of our findings among those with documented functional limitations. We assess statistical significance at the .05, .01, and .001 *p*-values and include 95% confidence intervals for ease of interpretation.

## Results

Table 1 shows the descriptive summary sample statistics. The care recipients, on average, report 10.71 depressive symptoms, as measured by the CESD scale.

**Table 1.** Descriptive Statistics of Care Recipients (HEPESE, Wave 7, 2010–2011; *n* = 760).

Variable	Mean (%)	(SD)	Count	Min	Max
Depressive symptoms	10.71	9.16		0	49
Sources of care					
Self care	45		339		
Family care	67		512		
Non family care	22		168		
Age	85.93	3.96		79	102
Female	64		483		
US born	56		422		
Married	35		263		
Total IADL count	5.16	3.39	3,925	0	10

Regarding care, 45% of respondents manage their own self-care, while a majority (67%) rely on at least one family member for care. A smaller proportion (22%) receives care from non-family sources.

The mean age of care recipients is 85.93 years, ranging from 79 to 102 years. The sample is predominantly female (64%) and native-U.S. born (56%). 35% of care recipients are married. Overall, the care recipients report an average score of 5.16 on the IADLs (ranging from 0 to 10). This score reflects moderate challenges in performing IADLs among the care recipients in this study.

Table 2 presents the results from our regression analysis. In Model 1, all three sources of care show significant associations with care recipients’ depressive symptoms. Relying on self-directed care was linked to significantly lower depressive symptoms (*b* = −4.41, 95% CI [−5.87, −2.94], *p* ≤ .001). Similarly, family care was associated with reduced depressive symptoms (*b* = −1.98, 95% CI [−3.49, −0.46], *p* ≤ .05). In contrast, non-family care was related to increased depressive symptoms (*b* = 4.03, 95% CI [2.52, 5.54], *p* ≤ .001). Among other covariates, being female (*b* = 2.48, 95% CI [1.09, 3.87], *p* ≤ .001) and being married (*b* = −1.67, 95% CI [−3.10, −0.24], *p* ≤ .05) were also significantly associated with depressive symptoms.

**Table 2.** OLS Regression Results: Source of Care on Care Recipient CESD Depressive Symptoms.

	Model 1			Model 2			Model 3		
	Care source			Care source + Total IADL			Only among those with IADL disability		
Variable	*b*	95% CI	*t*	*b*	95% CI	*t*	*b*	95% CI	*t*
									
Care recipient age	−0.08	[−0.24, 0.08]	−0.93	−0.19*	[−0.35, −0.03	−2.3	−0.20*	[−0.37, −0.02]	−2.24
Female	2.48***	[1.09, 3.87]	3.5	2.00**	[0.64, 3.36]	2.89	1.96*	[0.44, 3.48]	2.53
US born	−1.28*	[−2.51, −0.06]	−2.05	−0.95	[−2.15, 0.25]	−1.56	−0.96	[−2.26, 0.35]	−1.43
Married	−1.67*	[−3.10, −0.24]	−2.29	−1.36	[−2.76, 0.03]	−1.92	−1.69*	[−3.26, −0.13]	−2.12
Self-care	−4.41***	[−5.87, 2.94]	−5.92	−1.52	[−3.17, 0.14]	−1.8	−1.36	[−3.12, −0.41]	−1.51
Family care	−1.98*	[−3.49, −0.46]	−2.55	−2.54***	[−4.02, −1.05]	−3.35	−2.49**	[−4.15, −0.83]	−2.94
Non family care	4.03***	[2.52, 5.54]	5.22	2.84***	[1.33, 4.35]	3.68	2.81***	[1.24, 4.38]	3.51
Total IADL count				0.83***	[0.58, 1.07]	6.69	0.84***	[0.56, 1.13]	5.85
Constant	19.40**	[5.23, 33.57]	2.69	24.03***	[10.19, 37.88]	3.41	24.64**	[9.77, 39.50]	3.25
*N*	760			760			664		

* p ≤ .05. **p < .01. ***p ≤ .001.

In Model 2, the relationship between self-directed care and depressive symptoms was no longer significant; however, family care (*b* = −2.54, 95% CI [−4.02, −1.05], *p* ≤ .001) and non-family care (*b* = 2.84, 95% CI [1.33, 4.35], *p* ≤ .001) remained significant. Additionally, the total IADL count showed a positive relationship with depressive symptoms (*b* = 0.83, 95% CI [0.58, 1.07], *p* ≤ .001).

Finally, Model 3, restricted to respondents with at least one IADL disability, confirms the robustness of our findings regarding sources of care. Family care (*b* = −2.49, 95% CI [−4.15, −0.83], *p* ≤ .01) continues its association with reduced depressive symptoms, while non-family care (*b* = 2.81, 95% CI [1.24, 4.38], *p* ≤ .001) continues to be associated with increased depressive symptoms. Age (*b* = −0.20, 95% CI [−0.37, −0.02], *p* ≤ .05) and being female (*b* = 1.96, 95% CI [0.44, 3.48], *p* ≤ .05) remain significant predictors of depressive symptoms.

## Discussion

Our research investigates how the sources of informal care are associated with care recipients’ depressive symptoms. Although previous research has examined the impact of long-term caregiving on the mental well-being and reported depressive symptoms of caregivers (Schulz et al., 2020), the relationship between caregiver type and care recipients’ mental health remains understudied. By examining how care recipients’ depressive symptoms vary by caregiver relationship, our study provides crucial insights into an overlooked aspect of the caregiving dynamic.

Our study found that having informal family support for IADL disabilities is related to lower depressive symptoms in older adult care recipients. Older adults who received care from at least one family caregiver had significantly lower CESD scores than those who did not. This relationship remained significant even after controlling for the level of disability, proxied by number of IADL disabilities, while limiting the analysis to older adults with at least one IADL disability. This analytic approach necessarily treats family caregiving as a unified category. However, without more granular data distinguishing between caregiver roles (e.g., spouse vs. adult child), we are unable to examine how variations in relationship type may shape care recipients’ depressive symptoms. Future research should aim to disaggregate these caregiving roles to better capture their differential impact.

These results diverge from previous research using data from the longitudinal Health and Retirement Study, which found that activating care resources increased depressive symptoms (Lin & Wu, 2011). While earlier studies often combined informal support sources and found no variation in depressive symptoms by care source (Lin & Wu, 2011; Taylor & Lynch, 2004), our analysis reveals distinct patterns: family care appears protective against depressive symptoms, while non-family care is associated with increased symptoms. This difference may reflect our focus on Mexican Americans, versus previous studies’ reliance on nationally representative samples with limited Hispanic representation. Cultural expectations for family care among Mexican Americans may create a preference for family caregiving, making reliance on non-family caregivers a risk factor for depressive symptoms (Angel et al., 2004). Taken together, our results suggest that the source of care is a significant factor in depressive symptoms among older adults, emphasizing the need for policies that support family caregiving. While family-based caregiving is often seen as the preferred model, it can also create tension when some family members are unable or choose not to provide care.

Interventions that support family caregiving should also account for these dynamics by encouraging communication, promoting fair division of care, and offering resources that help families manage conflict in healthy ways. Policy initiatives at multiple levels could enhance support for family caregivers. Nationally, interventions such as support groups, counseling, and skills training would provide caregivers with crucial resources and community connections. Furthermore, national campaigns promoting paid family leave programs and insurance coverage for caregiving expenses (e.g., home care, necessary medical equipment) could help address the financial, mental, and physical challenges caregivers face (Stall et al., 2023).While payment of family caregiver is not available nationally through Medicare, some states offer programs to pay family caregivers through Medicaid and other state programs (Reckrey et al., 2025). Expansion of these programs could potentially allow family caregivers to take the role of non-family caregivers and in turn, be associated with fewer depressive symptoms in care recipients. Improved support during discharge planning and consistent access to mental health resources throughout the caregiving process could also help alleviate caregiver burnout. Workplace policies, including flexible work arrangements and extended family leave benefits, are also essential for maintaining work-life balance among caregivers.

While we advocate for strengthening family-based caregiving structures, we also acknowledge the complex moral terrain this occupies. When family members are unable or unwilling to provide care, societal narratives may wrongly frame this as neglect or abandonment, further stigmatizing caregivers and recipients alike (Cantu & Aranda, 2023). Future policy should support caregiving as a shared public responsibility rather than exclusively a private familial duty.

## Limitations

This analysis contributes to the scientific literature on caregiving in important ways. We are part of a growing literature examining how caregiving relationships affect outcomes of care recipients and specifically focus on Mexican Americans as a distinct segment of the U.S. population. Despite our contributions, there are limitations to the generalizability and approach of our analysis. Our analysis focuses solely on primary caregivers responsible for ADL care; however, recent studies indicate that the primary caregiver label may not capture the experiences of a large segment of informal caregivers (Hu et al., 2023). Additionally, we excluded non-family caregivers due to missing information on critical covariates such as relationship quality and care intensity, which limited our ability to make meaningful comparisons. Including non-family and paid professional caregivers in future studies could provide a more comprehensive picture of caregiving dynamics across diverse support networks. Although we are unable to assess the role of multiple caregivers in this study, our findings show that older adults who rely on non-family members as primary caregivers for ADL needs experience higher depressive symptoms, which appear to be unaffected by the size of the caregiving network. Indeed, reliance on non-family caregivers may correlate negatively with the size of the caregiving network (Angel et al., 2004). Additionally, our results rely on cross-sectional data limiting causal interpretations; we cannot rule out the possibility that higher depressive symptoms in care recipients might lead family members to prefer delegating caregiving responsibilities to non-family members. Little research has explored transitions out of caregiving roles by family members or the selection of new caregivers. Our findings suggest a need for future studies to examine the bidirectional relationship between caregiver selection and care recipient mental health. Further qualitative research is necessary to examine directional relationships for care and depressive symptoms.

## Conclusion

Our findings show that older Mexican American adults who reported being primarily responsible for their own IADL/ADL needs had significantly fewer depressive symptoms, compared to those receiving care from nonfamily members and reporting significantly higher depressive symptoms. These results align with the burden hypothesis, suggesting that older adults who receive care outside the family may experience increased depressive symptoms due to feeling burdensome, even when controlling for disability levels.

The results point to the need for interventions that enhance caregiving autonomy and support from family members in managing disabilities and potentially reducing depressive symptoms. For instance, strategies such as adaptive technologies and home modifications can support care recipients in maintaining their independence. Expanding patient education programs for both care recipients and caregivers can improve communication with healthcare providers and reduce caregiver burden. Such interventions, which prioritize autonomy and positive family involvement, hold promise for improving mental health outcomes for both caregivers and care recipients (Fried et al., 2005).
